# Comparisons of case-selection approaches based on allele sharing and/or disease severity index: application to the GAW14 simulated data

**DOI:** 10.1186/1471-2156-6-S1-S103

**Published:** 2005-12-30

**Authors:** Chunyu Liu, L Adrienne Cupples, Josée Dupuis

**Affiliations:** 1 Department of Neurology, Boston University School of Public Health, Boston, Massachusetts, USA; 2 Department of Biostatistics, Boston University School of Public Health, Boston, Massachusetts, USA

## Abstract

For mapping complex disease traits, linkage studies are often followed by a case-control association strategy in order to identify disease-associated genes/single-nucleotide polymorphisms (SNPs). Substantial efforts are required in selecting the most informative cases from a large collection of affected individuals in order to maximize the power of the study, while taking into consideration study cost. In this article, we applied and extended three case-selection strategies that use allele-sharing information method for families with multiple affected offspring to select most informative cases using additional information on disease severity. Our results revealed that most significant associations, as measured by the lowest *p*-values, were obtained from a strategy that selected a case with the most allele sharing with other affected sibs from linked families ("linked-best"), despite reduction in sample size resulting from discarding unlinked families. Moreover, information on disease severity appears to be useful to improve the ability to detect associations between markers and disease loci.

## Background

Linkage analyses are often the first step in mapping genes for complex traits. However, such methods typically implicate broad regions of the genome, and identifying causal genes remains a challenge. Association methods, which rely on linkage disequilibrium (LD) information, have a much better resolution. Therefore, one may want to follow a linkage peak with a case-control study to identify the exact causal variants in a region implicated by linkage analysis. To reduce the cost and increase the power of detecting a disease-marker association, different strategies have been exploited previously to identify genetically "loaded" individuals by choosing subjects with history of disease [1], a more severe form of disease [2], or early onset of disease [3] to increase the chance of detecting genetic risk factors in a population. Fingerlin et al. [4] proposed four strategies for selecting cases from families with multiple affected siblings to use in a case-control design in the search of disease susceptibility genes. In this paper, we apply and extend their methods to select the most informative cases using additional information on disease severity. Our goal is to choose individuals who maximize the expected difference in allele frequency between cases and controls.

## Methods

### Sample population and study design

An initial nonparametric genome scan was conducted in each of the 4 populations separately on a replicate selected at random (replicate 71), resulting in two regions linked to a simulated behavioral disorder. The first region was identified in the Karangar population on chromosome 9 at marker C09R0765; the second peak was located on chromosome 1 at marker C01R0052 in the Danacaa population. Both of these regions were worthy of more detailed scrutiny in the search for disease susceptibility loci. Both populations comprised 100 nuclear families with multiple affected offspring. Once regions linked to the trait were identified, 34 additional single-nucleotide polymorphisms (SNPs) were included under each of the linkage peaks: packets 417 and 418 under the chromosome 9 linkage peak and packets 28 and 29 under the linkage peak on chromosome 1. Allele-sharing probabilities were computed and a SNP genome scan analysis was carried out, with 91 SNP markers on chromosomes 9 and 95 SNP markers on chromosome 1. The additional packets of SNPs came with 50 controls for each replicate. We "collected" a sample of 100 unrelated controls by randomly selecting two replicates (71 and 35). The controls were not matched to any population because population admixture was not introduced into the simulations (allele frequencies are the same across all populations).

The disease of interest was a simulated behavioral disorder. In addition, presence or absence of 12 behavioral characteristics that may be related to the disease were also provided. We constructed a "severity" index for each individual by counting the number of characteristics present. In some instances we used this severity index in our case selection.

### Case-selection strategies

Fingerlin et al. [4] compared three case-selection strategies that use allele-sharing information with the standard strategy that selects a single individual from each family at random. In our study, we extended these strategies by using additional information on disease severity.

We considered the following 6 case-selection strategies to choose one case per sibship: i) a randomly selected case (all-random, or AR); ii) a case showing most identity-by-descent (IBD) sharing with other affected sibs (all-best, or AB); iii) a case with the most severe disease, i.e., with the highest severity index (all-most-severe, or AMS); iv) a randomly selected case per linked family (nonparametric linkage (NPL) LOD ≥ 0) (linked-random, or LR); v) a case with the most IBD sharing with other affected sibs from linked families only (linked-best or LB); vi) a case with the most-severe disease chosen from linked families (linked-most-severe, or LMS).

### Selecting families on the basis of multipoint linkage information

The software MERLIN [5] was used to calculate the Kong and Cox NPL LOD score for the entire sample [6]. The 34 additional genotyped SNPs were integrated into the map on both chromosomes 1 and 9. The "-npl" options was used to obtain summary LOD scores at each marker location. The "-perFamily" option along with "-npl" was used to obtain specific LOD scores for each family. We defined a family as showing evidence for linkage if the pedigree LOD score at the peak marker was ≥ 0. In this definition, families with less sharing than expected under no linkage were excluded.

### Selecting one sib with the most evidence for sharing with other affected sibs

We used the software MERLIN to calculate the amount of allele sharing between each pair of affected individuals in a family at each SNP marker under the linkage peak. The individual(s) with the most allele sharing obtained the highest "case-score" statistics in each family. There were 38 SNP markers under the linkage peak on chromosome 1 and 9, respectively. Therefore, each affected individual obtained 38 "case-scores". If there were two or more sibs with the same allele sharing score or the same severity, one of them was randomly selected. To minimize chance results from a specific random selection, 100 iterations were carried out to obtain an average test statistic.

### Test statistics

Allele frequencies were calculated on selected cases and controls for 38 SNPs on chromosomes 1 and 9. For each SNP, a chi-square test was performed to determine if there was a significant difference in allele frequencies between the selected cases and controls. The averaged chi-square statistics over 100 iterations were used to compute *p*-values for different case-selection strategies; *p*-values < 0.05 were considered statistically significant.

## Results

### Description of sibships and severity score index in both population

Table 1 describes the sibships in Danacaa and Karangar population. Most nuclear families had exactly two affected siblings. The Karangar included more families with multiple affected (≥ 2) individuals. The severity score index in unaffected and affected subjects (linked and unlinked) is summarized in Table 2. As expected, affected individuals had a much higher severity score than unaffected; however, individuals from linked and unlinked families had similar severity scores.

### Linkage analysis and IBD state calculation

After the additional 34 SNPs were integrated into the map, the peak NPL LOD score of 4.75 was found at marker B01T0559 at 168.36 cM on chromosome 1 and of 7.47 was found at marker B09T8335 at 5.45 cM on chromosome 9.

The most informative affected individuals were selected on the basis of linkage, allele sharing, and severity (see Methods). In both populations, AR, AB, and AMS include one affected individual from each of the 100 families. The LR, LB, and LMS selection have sample sizes of 75 and 73 for the Danacaa and Karangar populations respectively, which correspond to the number of linked families.

### Difference in allele frequencies on each marker location

**Figure 1:**
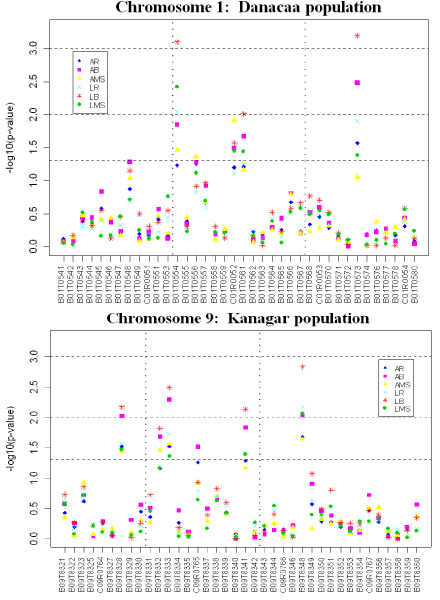
**Results of chi-square test to detect differences in allele frequency between cases and controls using six case-selection strategies**. The six case-selection strategies are: i) a randomly selected case (AR); ii) a case showing most IBD sharing with other affected sibs (AB); iii) a case with the most severe disease (AMS); iv) a randomly selected case per linked family (LR); v) a case with most IBD sharing with other affected sibs from linked families only (LB); vi) a case with the most-severe disease chosen from linked families only (LMS). The horizontal dashed lines on both figures denote *p*-values of 0.05, 0.01, and 0.001, respectively. The regions between the vertical dashed lines represent the haplotype region containing the disease locus.

Figure 1 displays chi-square test results (negative logarithm base 10 of the *p*-value) to detect difference in allele frequencies for 38 SNPs between cases and controls using the 6 strategies in Karangar and Danacaa populations. The horizontal dashed lines on both figures denote *p*-values of 0.05, 0.01, and 0.001, respectively. The regions between the vertical dashed lines represent the haplotype region containing the disease locus on each chromosome. The data were generated from disease susceptibility haplotypes; thus, a single causal SNP is not available.

Both figures reveal that smallest *p*-values were obtained from the LB selection strategy, despite reduction in sample size from using individuals in linked families only. Three SNPs (one on chromosome 1 and two on chromosome 9) that yielded significant association reside outside the haplotype regions. These may be type I errors. The figures also indicate that using information on disease severity appears to improve the ability for detecting association between markers and disease loci. On chromosome 1, all significant SNPs using the AMS selection were within the haplotype region.

## Discussion

Our results indicate that more efficient case-control study designs could be obtained by selecting cases with the most evidence for allele sharing in addition to linkage information, and possibly incorporating information on disease severity. Despite the decrease in sample size resulting from exclusion of unlinked families, effect size, as measured by the chi-square statistic comparing allele frequency in the cases and controls, can be increased by using this information.

We initiated our study to identify disease-associated SNPs in those two chromosome regions based solely on results from the initial genome scan without more information about the data or how the disease locus was simulated. Among our 6 selection strategies, the LB method yielded smallest *p*-values on both chromosomes, despite a decrease in sample size resulting from exclusion of unlinked families. Our results also indicated that most of the SNPs that had significant *p*-values resided within the simulated haplotype regions. The extension of using information on disease severity seems to improve the precision of detection, since no type I errors were observed using AMS on chromosome 1. In Figure 1, none of the significant *p*-values would remain significant after adjusting for testing multiple SNPs. However, the sample size used in this study is very small (*N *≤ 100 cases). Therefore, it is likely to be a powerful strategy for larger sample sizes.

The strategies in our study are most useful if there are causal SNPs and markers in LD with these SNPs, or the causal variants themselves have been genotyped. In the Genetic Analysis Workshop 14 data, disease status was simulated from haplotypes and the causal variants themselves were not available. On chromosome 1, the maximum estimated *r*^2 ^(pair-wise) value was only 0.03, which indicated virtually no LD was present. On chromosome 9, the two largest *r*^2 ^values were 0.87 (between B09T8338 and B09T8339) and 0.56 (between B09T8337 and B09T8338), respectively, indicating a region with higher LD values. However, neither of those SNPs reached statistical significance at the 0.05 level. In the absence of knowledge of the LD pattern between the tested SNPs and the causal SNP, it is difficult to make general conclusions from the current study. Nevertheless, some interesting results emerged from our analyses. Extension of the case selection strategy to take haplotype information into consideration may be more successful. We are exploring such extensions.

## Abbreviations

AB: All-best

AMS: All-most-severe

AR: All-random

IBD: Identity by descent

LB: Linked-best

LD: Linkage disequilibrium

LMS: Linked-most-severe

LR: Linked-random

NPL: Nonparametric linkage

SNP: Single-nucleotide polymorphism

## Authors' contributions

CL contributed to the concept and study design, data analysis and interpretation, and was involved in drafting the manuscript. LAC contributed to concept and study design. JD contributed to the concept and study design, data analysis, and interpretation. All authors read and approved the final manuscript.

## Figures and Tables

**Table 1:** Description of sibships

Sibship size	*n *sibs	*n *affected	*n *unaffected
Danacaa population			
0	0	0	10
1	0	0	14
2	10	85	16
3	12	12	21
4	15	2	11
5	19	1	12
6	13	0	8
7	13	0	8
8	5	0	0
9	13	0	0
Total	100	100	100
			
Karangar population			
0	0	0	0
1	0	0	9
2	0	54	19
3	0	34	18
4	17	9	19
5	15	3	12
6	24	0	7
7	16	0	8
8	7	0	7
9	7	0	1
10	12	0	0
11	1	0	0
12	1	0	0
Total	100	100	100

**Table 2:** Severity score index in unaffected and affected (linked and unlinked) in Danacaa and Karangar population

	Danacaa population		Karangar population	
		
	Sample size	Severity score mean (Q3-Q1)*	Sample size	Severity score mean (Q3-Q1)*
All unaffected	317	1.15 (2)	239	1.27 (2)
All affected	220	5.47 (1)	222	7.41 (1)
Linked	166	5.59 (1)	161	7.34 (1)
Unlinked	54	5.09 (1)	61	7.60 (1)
Control	100	0.70 (1)	100	0.70 (1)

*Q3-Q1: interquartile range
